# Comparative analysis of machine learning versus traditional method for early detection of parental depression symptoms in the NICU

**DOI:** 10.3389/fpubh.2024.1380034

**Published:** 2024-05-28

**Authors:** Fatima Sadjadpour, Niyousha Hosseinichimeh, Vida Abedi, Lamia M. Soghier

**Affiliations:** ^1^Department of Industrial and Systems Engineering, Virginia Polytechnic Institute and State University, Blacksburg, VA, United States; ^2^Department of Public Health Sciences, Penn State University, College of Medicine, Hershey, PA, United States; ^3^Department of Neonatology, Children’s National Hospital, Washington, DC, United States; ^4^The George Washington University School of Medicine and Health Sciences, Washington, DC, United States; ^5^Children’s Research Institute, Children’s National Hospital, Washington, DC, United States

**Keywords:** parental depression, neonatal intensive care unit, NICU, screening system, machine learning, logistic regression

## Abstract

**Introduction:**

Neonatal intensive care unit (NICU) admission is a stressful experience for parents. NICU parents are twice at risk of depression symptoms compared to the general birthing population. Parental mental health problems have harmful long-term effects on both parents and infants. Timely screening and treatment can reduce these negative consequences.

**Objective:**

Our objective is to compare the performance of the traditional logistic regression with other machine learning (ML) models in identifying parents who are more likely to have depression symptoms to prioritize screening of at-risk parents. We used data obtained from parents of infants discharged from the NICU at Children’s National Hospital (*n* = 300) from 2016 to 2017. This dataset includes a comprehensive list of demographic characteristics, depression and stress symptoms, social support, and parent/infant factors.

**Study design:**

Our study design optimized eight ML algorithms – Logistic Regression, Support Vector Machine, Decision Tree, Random Forest, XGBoost, Naïve Bayes, K-Nearest Neighbor, and Artificial Neural Network – to identify the main risk factors associated with parental depression. We compared models based on the area under the receiver operating characteristic curve (AUC), positive predicted value (PPV), sensitivity, and F-score.

**Results:**

The results showed that all eight models achieved an AUC above 0.8, suggesting that the logistic regression-based model’s performance is comparable to other common ML models.

**Conclusion:**

Logistic regression is effective in identifying parents at risk of depression for targeted screening with a performance comparable to common ML-based models.

## Introduction

Postpartum depression (PPD) can occur in women after childbirth for up to one year, affecting around 15% of mothers, and is the most common complication of childbirth (1). Neonatal intensive care unit (NICU) admission is a stressful experience for parents and together with prematurity are well known risk factors for PPD. Multiple studies have determined that the incidence of PPD in parents whose infants are admitted to the NICU is approximately 40–45%, which is considerably higher than the 15% risk among general birthing population (2, 3). Therefore, early detection of PPD at critical times during admission and discharge through screening programs can play a significant role in preventing the negative consequences for the family and child (4). Given the importance of early diagnosis of depression symptoms, multiple NICUs have developed and implemented screening programs for PPD in the NICU (4–6). Early identification of depression symptoms in parents is crucial to mitigate adverse effects such as infant neurodevelopmental delays (7). However, the current screening process is both expensive and time-consuming, requiring a tracking system that could span multiple healthcare settings.

Having a predictive model to identify parents at risk of developing postpartum depression can assist in prioritizing those in need of screening. Prior research has focused on training machine learning (ML) models to predict postpartum depression (8–19). A review of these studies revealed several significant predictors, including age, education, marital status, income, ethnicity, lifetime depression, depression during pregnancy, anxiety, smoking, mode of delivery, gestational age, APGAR score (appearance, pulse, grimace, activity, and respiration), BMI (body mass index), and history of antidepressant use (10). Although ML models have been used to predict postpartum depression, no study has applied ML to predict postpartum depression of NICU parents. There is only one study that has utilized logistic regression to investigate the risk factors associated with parental depression symptoms at NICU (20) and found that higher levels of parental stress, older gestational age, and lower levels of social support contribute to parental depression symptoms at NICU (20). However, it is worth noting that this study has yet to present performance metrics for the model, which are essential for facilitating a comprehensive comparison with other predictive models. Notably, the study also did not employ segmentation of the data into training and testing sets, a practice pivotal for evaluating the model’s performance using unseen testing data. The absence of such data partitioning is a common feature of preliminary investigations which may raise concerns about the model’s ability to generalize beyond its training data.

This study contributes to the existing literature in three distinct ways. Firstly, it pioneers the application of machine learning (ML) approaches to NICU data to comprehensively investigate factors predicting postpartum depression among NICU parents. A central objective is to discern whether ML methodologies surpass the predictive capabilities of the traditional Logistic Regression (LR) model. Secondly, the study employs rigorous methodology by dividing the dataset into distinct training and testing sets. Multiple performance measures are reported to systematically compare and assess the efficacy of eight ML models on previously unseen data (testing dataset). The study also undertakes data imputation and parameter optimization, ensuring robustness and reliability of the findings. Thirdly, this research enhances the existing logistic regression model by incorporating two pivotal variables, namely anxiety level and self-efficacy. Moreover, improvements in data preprocessing steps contribute to a more nuanced understanding of the intricate relationship between these variables and parental depression in the NICU context.

## Methodology

### Study population

This study is based on the clinical data collected from three hundred parent-infant dyads who were anticipating discharge from the level IV NICU at Children’s National Hospital in Washington, DC, between January 2016 and February 2017 as part of the giving parents support (GPS) trial (20). This level IV NICU provides care to complex term and preterm infants and offers parental support services such as parental education, support groups, social work, and mental health services. Inclusion criteria were one parent (either mother or father) aged ≥18 years who were self-identified as the primary caregiver for the next year. Questionnaires were used to collect data about parent and infant characteristics, and validated screening tools [Center for Epidemiological Studies-Depression scale 10 (CES-D-10)] were given prior to discharge to determine incidence of depression symptoms. The study was reviewed and approved by the CN Institutional Review Board, and it was registered with https://clinicaltrials.gov (NCT02643472) (20).

### Data elements

A total of eighteen independent variables, including demographics of the parents, health profile of the infants, hospital stay, and various stress levels and social support network of the family, were used in this study. More specifically, parents’ demographic characteristics included race, age, gender, education, relationship status, having other children at home, working status prior to having the NICU infant, and current working status. Stress and anxiety were assessed using the following scales: Perceived Stress Scale (PSS-10), which assesses general stress. Parental Stress Scale (PSS) which measures parental stress regarding their new parenting role. Parental Stress Scale at NICU (PSS NICU) which evaluates NICU-specific stress after admission to the NICU and is based on infant appearance, NICU sights and sounds, parental role alterations, and parent relationships with staff. Multidimensional Scale of Perceived Social Support MSPSS was used to assess the parents’ perception of social support given to them by significant others, family, and friends. Perceived Maternal Parenting Self-efficacy PMPSE measured the parent’s belief in their ability to provide sufficient care for the infant. STAI Y-1 (state anxiety scale) and STAI Y-2 (trait anxiety scale) assessed the current anxiety state of parents and parents’ baseline anxiety characteristics. Infant characteristics were included as the independent variables such as infant gender in NICU, birth weight, birth weight < 1,500 grams, gestational age (weeks), and length of stay (LOS) in NICU (days) (20). The primary outcome measure was depression symptoms of each parent which was collected by the 10-item questionnaire of Center for Epidemiological Studies Depression Scale (CESD-10) and a total score of ≥10 indicated an elevated depression symptom.

### Data preprocessing and imputation

#### Data preprocessing

**Figure 1:**
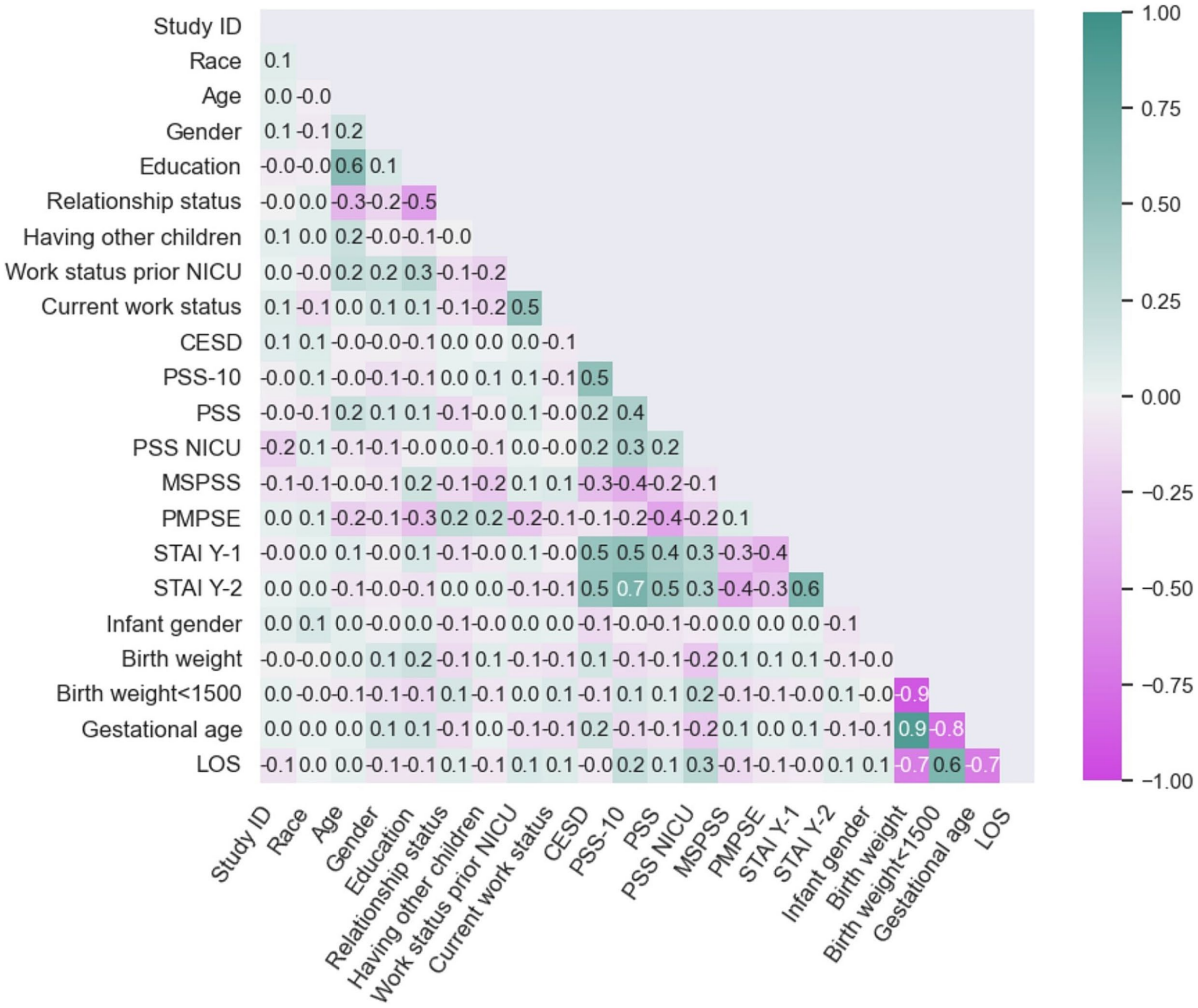
Correlation plot.

Multicollinearity was addressed by examining the correlation matrix presented in Figure 1, which enabled the identification of predictors exhibiting high correlation. Variables with a correlation exceeding 0.8 or falling below −0.8 were deemed highly correlated. To mitigate the impact of multicollinearity on the results, the variables representing birth weight and birth weight less than 1,500 grams were excluded from further analysis, given their significant correlation with gestational age.

#### Missing data and imputation

Some independent variables exhibited missing values that required addressing before analysis commenced. The number of missing values per variable was as follows: PSS NICU: 9 (3%), PMPSE: 9 (3%), MSPSS: 6 (2%), PSS: 5 (1%), STAI Y-2: 5 (1%), STAI Y-1: 4 (1%), PSS-10: 4 (1%). To address this, we implemented an imputation criteria approach. The highest number of missing values per participant was seven, which indicated a lack of response to all seven surveys. The three participants with seven missing values were excluded (*n* = 3, 1%). Imputation was applied for participants with less than two missing values (*n* = 18), encompassing 15 participants with only one missing value, and 3 participants with two missing values. After evaluating the distribution of variables with low missing rates and determining their non-normal distribution, we chose median imputation as the preferred technique. Median imputation is often favored for handling skewed data distributions due to its reduced sensitivity to outliers in comparison to mean imputation techniques. This decision was specifically made to address the conditions of low missing rates (6%) and non-normal distributions, ensuring a robust imputation approach for the dataset (21). Less than two missing values per patient for a total of eighteen patients (6%) were imputed using this strategy. The entire process of data cleaning, analysis, and the development of machine learning models was conducted in Python 3 using the Jupyter Notebook interface.

### Statistical analysis

A descriptive statistical analysis was performed to analyze the characteristics of the study population and identify the prevalence of depression symptoms among various groups. The cohort for this study included three hundred parent-infant pairs; after excluding three participants due to high missingness, a total of 297 parent-infant pairs were analyzed and included in the study. To ensure consistency with the past similar studies (20), a same stratifying strategy for birth weight categories, gestational age, and length of stay was employed during the analysis. Table 1 shows the demographic and clinical characteristics of the parents and their infant. Importantly, the variables presented in the following table did not have any missing values, reinforcing the robustness of our dataset and analysis. The unadjusted statistics presented in Table 1 reveal significant distinctions between the high-risk and low-risk groups in terms of infant gender (*p*-value = 0.02) and gestational age (*p*-value = 0.03), without accounting for the influence of other variables.

**Table 1:** Demographic and clinical characteristics of the parents and their infant.

		Total (*n* = 297)	High depression score (*n* = 135)	Low depression score (*n* = 162)	*p*-value
Parental demographic characteristics		Variables, *n* (%)			
Race	White/Caucasian	116 (39)	54 (40)	62 (38)	0.2517
	Black/African American	132 (44)	53 (39)	79 (48)	
	Asian	17 (6)	8 (6)	9 (6)	
	American Indian	8 (3)	5 (4)	3 (2)	
	Mixed race	24 (8)	15 (11)	9 (6)	
Age (years)	Mean ± SD	30 ± 6	30 ± 6	30 ± 7	0.6268
Gender	Female	264 (89)	121 (90)	143 (88)	0.8532
	Male	33 (11)	14 (10)	19 (12)	
Education	High school diploma or less	77 (26)	36 (27)	41 (25)	0.4018
	Trade/vocational training/some college	85 (29)	43 (32)	42 (26)	
	College/university degree or higher	135 (45)	56 (41)	79 (49)	
Relationship status	Married partner/spouse	159 (54)	70 (52)	89 (55)	0.9103
	Unmarried partner/spouse	87 (29)	42 (31)	45 (28)	
	Single	49 (16)	22 (16)	27 (16)	
	Divorced	2 (1)	1 (1)	1 (1)	
Having other children	Yes	169 (57)	77 (57)	92 (57)	1
	No	128 (43)	58 (43)	70 (43)	
Work status prior NICU infant	Yes	210 (71)	98 (73)	112 (69)	0.5252
	No	87 (29)	37 (27)	50 (31)	
Current work status	Yes, full time	114 (38)	50 (37)	64 (39)	0.6665
	Yes, part time	37 (12)	15 (11)	22 (14)	
	No	146 (49)	70 (52)	76 (47)	
Infants’ clinical characteristics					
Infant gender in NICU	Female	126 (42)	67 (50)	59 (36)	0.0252 *
	Male	171 (58)	68 (50)	103 (64)	
Birth weight < 1,500 gr	Yes	63 (21)	23 (17)	40 (25)	0.1185
	No	234 (79)	112 (83)	122 (75)	
Birth weight category (grams)	< 1,000	31 (10)	10 (7)	21 (13)	0.0974
	1,000–1,499	31 (10)	12 (9)	19 (12)	
	1,500–2,499	62 (21)	24 (18)	38 (23)	
	> 2,500	173 (58)	89 (66)	84 (52)	
Gestational age (weeks)	< 28	29 (10)	7 (5)	22 (13)	0.0353 *
	28–33	61 (21)	24 (18)	37 (23)	
	34–36	39 (13)	18 (13)	21 (13)	
	> 37	168 (57)	86 (64)	82 (51)	
Length of stay (days)	1–7	78 (26)	38 (28)	40 (25)	0.2321
	8–17	71 (24)	29 (21)	42 (26)	
	18–47	75 (25)	40 (30)	35 (21)	
	48–181	73 (25)	28 (21)	45 (28)	

^*^Statistically significant *p*-value.

### Model development

The refinement process of the 297 participants included in this study involved a random split into training (80%) and testing (20%) sets. Given the small sample size and complexities of predicting parental depression symptoms, this split ratio was considered appropriate to strike a balance between model performance and the robustness of the findings. The stratified sampling technique (22) was employed during this split to ensure a balanced distribution of samples between the training and testing subsets. For the final evaluation, 20% of the data was reserved for testing, while the remaining 80% was utilized in the cross-validation process. This involved dividing the 80% dataset into 10 folds, with the model undergoing training 10 times. Each iteration used a different fold as the test set (24 data points) and the remaining as the training data (213 data points), ensuring a robust learning experience. The assessed accuracy of the models is reported as the mean score across these 10 repetitions.

Eight diverse algorithms, namely Logistic Regression (LR), Support Vector Machine (SVM), Decision Trees, Random Forest (RF), Extreme Gradient Boosting (XGBoost), Naive Bayes (NB), and K-Nearest Neighbor (KNN), were implemented using the scikit-learn package in Python (23). Additionally, Artificial Neural Networks (ANN) were utilized through the Keras library in Python (24). Hyperparameter tuning using a combination of grid search, parallel processing and dropout regularization was conducted for ANN to identify optimal parameter combinations while monitoring corresponding learning curve to prevent overfitting issues.

**Figure 2:**
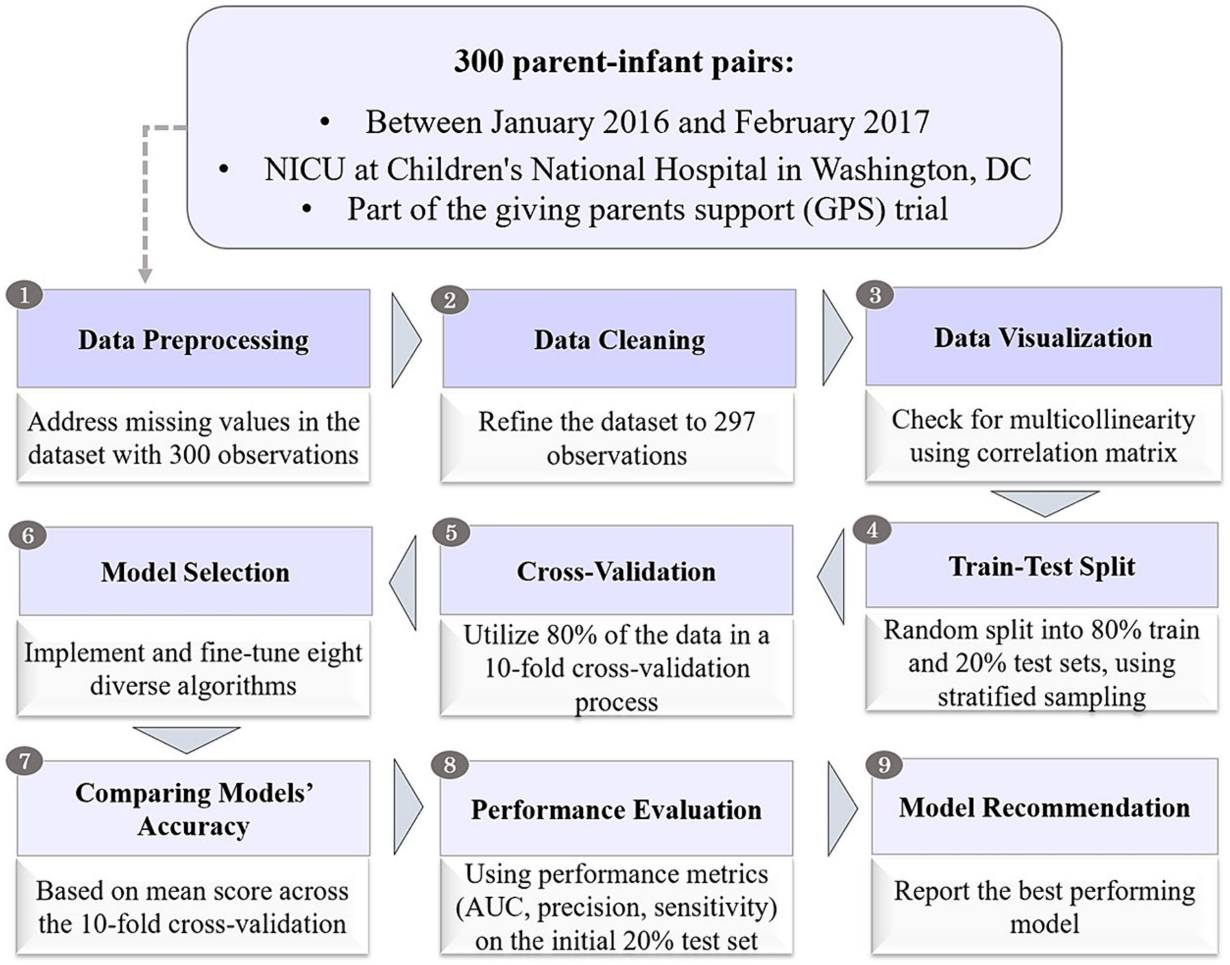
Process chart for model development.

Moreover, it is important to note that cross-validation was employed solely to obtain the mean accuracy of each ML algorithm. The actual performance metrics such as area under the receiver operating characteristic curve (AUC), precision (positive predicted value – PPV), sensitivity (recall), F-score, and in-depth analysis were implemented using the initial 20% of the test data, ensuring a comprehensive evaluation based on a separate, independent subset. A process chart of model development is provided in Figure 2.

## Results

### Logistic regression

In our study, we enhanced the performance of the existing logistic regression (LR) model, originally constructed on this dataset (20), by incorporating additional variables capturing perceived self-efficacy (PMPSE), STAI Y-1 (state anxiety scale) and STAI Y-2 (trait anxiety scale). Furthermore, we implemented a meticulous preprocessing procedure to address missing values. The summarized results in Table 2 displays the LR model’s outcomes, revealing PSS-10 (perceived stress scale), MSPSS (multidimensional scale of perceived social support), STAI Y-1 (state anxiety scale), infant female gender, and older gestational age (GA) as significant variables in predicting parental depression symptoms. Notably, our findings align with the outcomes of the previous study that employed logistic regression on this same dataset (20). This consistency underscores the robustness and reliability of our extended LR model.

**Table 2:** Logistic regression results for predictors of parental depression symptoms at NICU.

Variables	Coef.	Std. err.	*z*	*p* > |z|	[0.025]	[0.975]
Race	0.0993	0.1550	0.6408	0.5217	−0.2045	0.4032
Age	−0.0038	0.0328	−0.1153	0.9082	−0.0681	0.0605
Parent gender	−0.2878	0.5992	−0.4802	0.6311	−1.4622	0.8867
Education	−0.3261	0.2840	−1.1482	0.2509	−0.8828	0.2306
Relationship status	−0.2359	0.2636	−0.8950	0.3708	−0.7525	0.2807
Having other children	−0.5163	0.4043	−1.2770	0.2016	−1.3088	0.2762
Working prior NICU infant	0.3531	0.4980	0.7090	0.4783	−0.6230	1.3291
Currently working	−0.1749	0.3021	−0.5791	0.5626	−0.7670	0.4171
PSS-10^a^	0.1090	0.0354	3.0797	0.0021*	0.0396	0.1783
PSS^b^	−0.0457	0.0284	−1.6090	0.1076	−0.1015	0.0100
PSS NICU^c^	0.0032	0.0046	0.6948	0.4872	−0.0058	0.0122
MSPSS^d^	−0.0464	0.0168	−2.7590	0.0058*	−0.0794	−0.0135
PMPSE^e^	−0.0153	0.0194	−0.7869	0.4313	−0.0533	0.0228
STAI Y-1^f^	0.0610	0.0192	3.1783	0.0015*	0.0234	0.0986
STAI Y-2^g^	0.0339	0.0282	1.2034	0.2288	−0.0213	0.0892
Infant gender (female)	−0.9698	0.3598	−2.6952	0.0070*	−1.6750	−0.2645
Older gestational age	0.6299	0.2388	2.6382	0.0083*	0.1619	1.0978
LOS^h^	0.0353	0.2165	0.1632	0.8703	−0.3889	0.4596

*Statistically significant *p*-value.

a PSS-10: perceived stress scale.

b PSS: parental stress scale.

c PSS NICU: parental stress scale NICU.

d MSPSS: multidimensional scale of perceived social support.

e PMPSE: perceived maternal parenting self-efficacy.

f STAI Y-1: state anxiety scale.

g STAI Y-2: trait anxiety scale.

h LOS: length of stay.

### Training machine learning models

**Figure 3:**
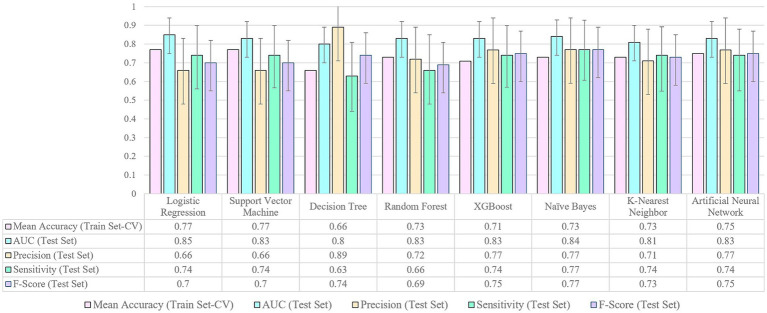
Performance metrics for machine learning models.

Models were trained on 80% of the dataset and then evaluated on the remaining 20% of the data. The actual value of performance metrics including area under the curve (AUC), precision, or positive predicted value (PPV), sensitivity or recall, and F-score are presented in Figure 3. Also, the 95% confidence interval of the performance metrics are shown as the error bars in Figure 3 and in more detail presented in Table 3.

**Table 3:** Confidence intervals of performance metrics for machine learning models.

Method	AUC (95% CI)	Precision (95% CI)	Sensitivity (95% CI)	*F*-score (95% CI)
Logistic regression	(0.75, 0.94)	(0.48, 0.83)	(0.56, 0.90)	(0.55, 0.82)
Support vector machine	(0.72, 0.93)	(0.50, 0.82)	(0.57, 0.90)	(0.55, 0.81)
Decision tree	(0.67, 0.92)	(0.73, 1.00)	(0.44, 0.81)	(0.59, 0.86)
Random forest	(0.73, 0.93)	(0.54, 0.88)	(0.48, 0.85)	(0.53, 0.82)
XGBoost	(0.71, 0.93)	(0.60, 0.92)	(0.57, 0.90)	(0.61, 0.87)
Naïve Bayes	(0.74, 0.93)	(0.60, 0.92)	(0.61, 0.93)	(0.64, 0.89)
K-nearest neighbor	(0.71, 0.91)	(0.54, 0.88)	(0.55, 0.89)	(0.58, 0.84)
Artificial neural network	(0.72, 0.92)	(0.60, 0.92)	(0.55, 0.88)	(0.61, 0.87)

In analyzing the performance metrics of the various models, several key observations emerge. The mean accuracy on the training set, as assessed through cross-validation, reveals that Logistic Regression and Support Vector Machine achieved relatively high accuracies at 0.77. However, it is crucial to consider additional metrics for a comprehensive evaluation. The AUC on the test set serves as a vital indicator of a model’s ability to discriminate between two classes of low and high depression risks, with values closer to 1 indicating better performance. Notably, Logistic Regression, Support Vector Machine, Naïve Bayes, Artificial Neural Network, Random Forest and XGBoost demonstrated competitive AUC values ranging from 0.83 to 0.85. Precision represents the accuracy of the model in identifying parents at risk of depression among those predicted as high-risk. A high precision indicates a low rate of false positives, meaning that when the model predicts a parent as high-risk, there is a high probability that they indeed have an elevated risk of depression. Decision Tree stands out with a high precision value of 0.89. Sensitivity, also called recall, measures the ability of the model to correctly identify parents who are truly at high risk of depression among all the parents who are at high risk. High sensitivity implies that the model is effective in capturing a significant portion of parents with a high risk of depression, minimizing the number of cases being missed. Naïve Bayes excels in sensitivity at 0.77, emphasizing its’ effectiveness in identifying positive cases despite a relatively lower mean accuracy. F-score is a metric that combines precision and sensitivity into a single score, providing a balanced assessment of a model’s performance in making accurate positive predictions while minimizing both false positives and false negatives. Naïve Bayes, XGBoost, and Artificial Neural Network demonstrate high F-score values ranging from 0.75 to 0.77, indicating a good model performance in terms of both precision and sensitivity. The choice of the optimal model should consider trade-offs between precision and sensitivity based on specific application goals —for instance, whether avoiding false alarms (high precision) or capturing as many true cases as possible (high sensitivity) or both is more critical in the context of predicting depression risk in parents of NICU infants.

**Figure 4:**
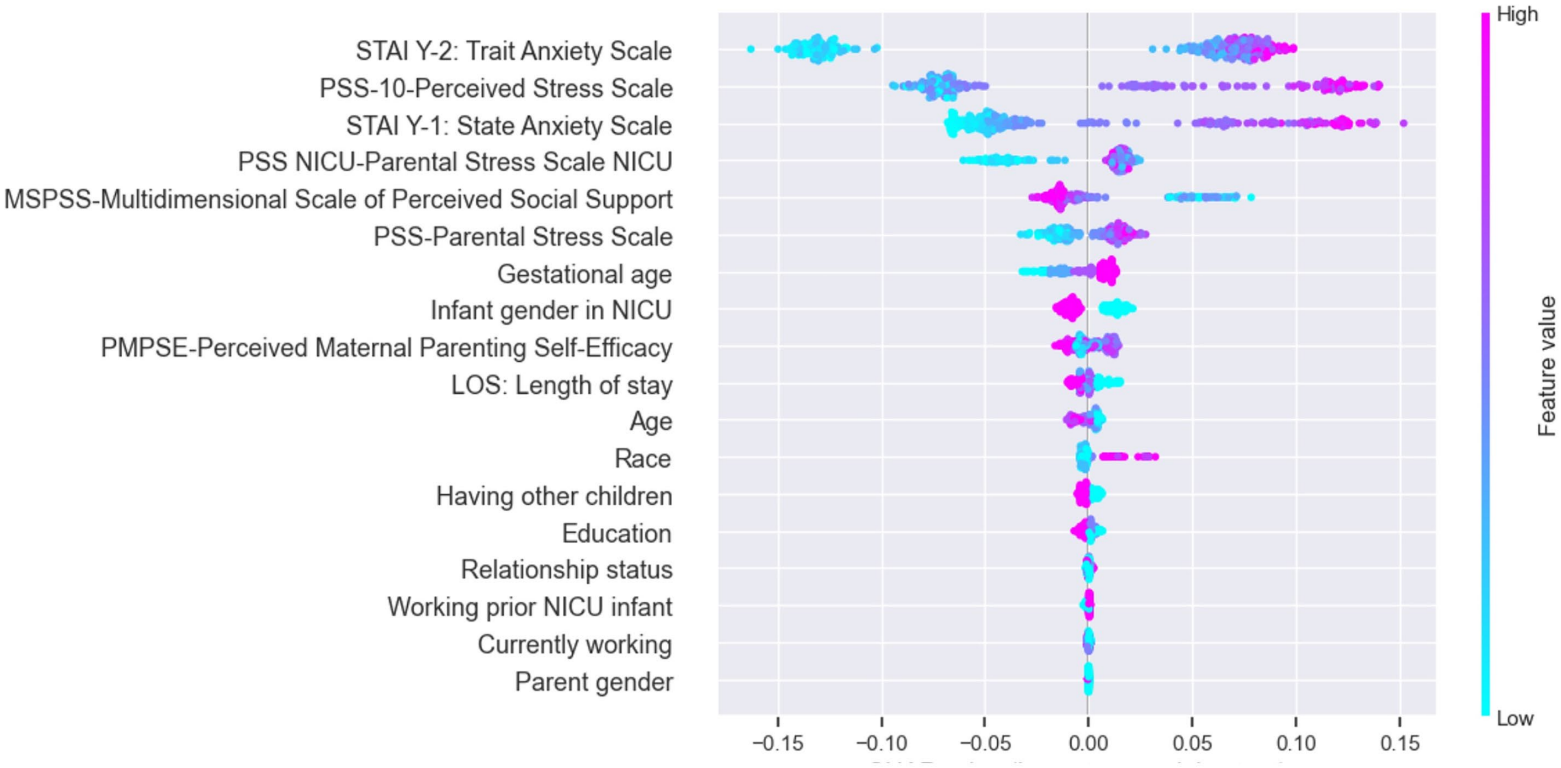
SHAP value presenting impact on model output (for output label “1”: high risk class).

Building on the discussion of trade-offs between performance metrics, the SHAP value analysis of variable importance in Figure 4 sheds light on the key contributors to predicting parental depression symptoms at NICU discharge. According to the Figure 4, the top five variables impacting the risk of depression are STAI-Y2 (trait anxiety scale), PSS-10 (perceived stress scale), STAI-Y1 (state anxiety scale), PSS-NICU (parental stress scale NICU), and MSPSS (multidimensional scale of perceived social support). These findings provide valuable insights into the specific variables driving the model’s predictions, reinforcing the significance of specific variables in predicting parental depression symptoms at NICU discharge. The SHAP analysis approach is specifically useful as it allows us to assess the extent of the impact of these variables on the prediction of our outcome (25). For example, Figure 4 pinpoints STAI-Y2 (trait anxiety scale), as the most important variable for parental depression estimation. When STAI-Y2 is “low” (blue), the log-odds of model predicting “high risk class” decreases by up to 0.15 units. Conversely when STAI-Y2 is “high” (pink), the log-odds of model predicting “high risk class” increases by up to 0.10 units.

### Comparison of logistic regression with other ML models

**Figure 5:**
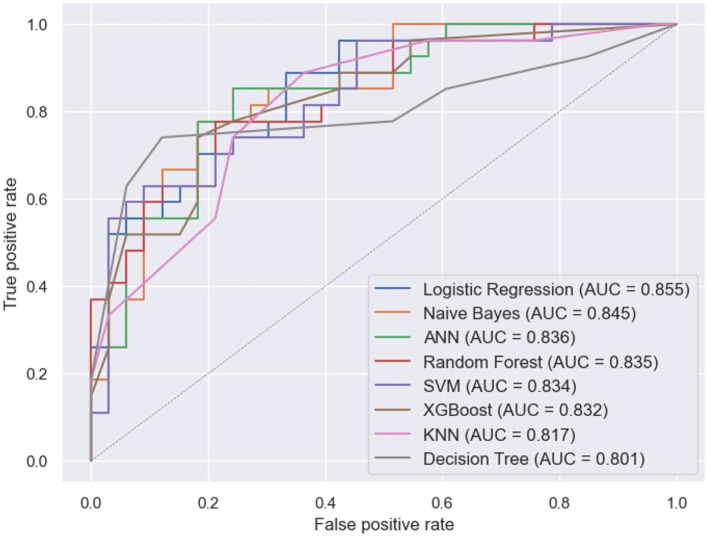
ROC curves for machine learning models.

Building upon the observation of overlapping confidence intervals in Figure 3, signifying comparable performance across models, it becomes evident that distinctions in sensitivity, precision, F-score, and area under the curve are not statistically significant. For instance, the sensitivity of the Naïve Bayes (0.77) surpasses that of logistic regression (0.74), yet falls within the confidence interval of logistic regression’s sensitivity (0.56–0.9), a trend echoed in other performance metrics (detailed confidence interval information is available in Table 3). Given these findings, it is clear that all models exhibit comparable performance statistics, with logistic regression standing out in Figure 5 by achieving the highest area under the curve (AUC). This consistency in performance, coupled with the superior interpretability of logistic regression, positions it as a preferable choice for predicting parental depression symptoms at NICU discharge.

## Discussion

In tackling the complexities inherent in forecasting the risk of parental depression upon NICU discharge, our study takes a comprehensive approach, aiming to identify and prioritize factors associated with this crucial outcome. We sought to establish the most effective predictive model by systematically comparing results obtained from various machine learning (ML) techniques and logistic regression (LR). While previous research in the domain of predicting parental mental health outcomes has delved into the application of ML models (8–19), the specific context of parental depression in the NICU remains underexplored, with only logistic regression studies to date (20). In the absence of conclusive evidence supporting ML’s superiority in predicting parental depression within the NICU, our study fills a critical gap by offering a rigorous comparison between ML techniques and logistic regression. This investigation emerges from a motivation to challenge the assumption that ML universally outperforms traditional methods, especially in the nuanced domain of parental mental health within the NICU. By providing empirical evidence and insights into the predictive efficacy of different methodologies, our research contributes to advancing the understanding of optimal prediction strategies in this unique healthcare context.

Building upon this motivation, our study endeavors to elevate the field by advancing beyond the limitations of the existing logistic regression study on parental depression in the NICU. We explore this uncharted territory by employing eight distinct machine learning (ML) models, each meticulously assessed and compared through comprehensive performance evaluations on previously unseen test data. This departure from conventional methodologies is facilitated by the implementation of a cross-validation technique, dividing the data into two subsets for model evaluation, ensuring robustness and applicability to real-world scenarios. Figure 3 presents a visual representation of our findings, encapsulating crucial performance measures such as accuracy, AUC, precision, sensitivity, and F-score. This not only facilitates an in-depth comparison of the models but also ensures the reproducibility of our results across different frameworks. Furthermore, our study enhances the existing paradigm by fine-tuning a previous model (20), incorporating additional independent variables such as state anxiety scale (STAI-Y1), trait anxiety scale (STAI-Y2), and perceived maternal parenting self-efficacy. This augmentation, coupled with refined preprocessing procedures, contributes to the evolution of predictive models in the NICU setting.

Our findings reveal that a higher level of perceived stress (PSS-10), lower perceived social support (MSPSS), and older gestational age (GA) significantly contribute to depression symptoms among parents of NICU infants. Importantly, our results align statistically with those reported in Soghier et al. (20). Additionally, we observed that parents with a female infant in the NICU face a higher risk of depression symptoms compared to parents of male infants. While the precise reasons for this gender difference remain elusive, analogous results have been noted in other studies, where the reported odds of depression are higher among mothers of female infants (26–28). These studies attribute this outcome to a potential preference for a male infant, suggesting societal influences. Limited evidence also suggests biological differences; mothers carrying a female fetus exhibit elevated levels of β-human chorionic gonadotropin. This indicates that hormonal changes, along with similar alterations, may provide a biological explanation for the impact of the child’s gender on postnatal depression (29, 30).

Building on these significant findings, our study introduces an additional layer of insight by delving into feature importance through SHAP analysis. By not exclusively relying on black box ML-based models, we were able to extract nuanced information about the contributors to parental depression symptoms in the NICU. Particularly noteworthy are the state anxiety scale (STAI-Y1) and trait anxiety scale (STAI-Y2), identified as crucial predictors. This finding emphasizes the importance of not only screening for depression but also for anxiety and social support as both naturally predict the onset of depression. While the connection between anxiety, social support, and depression is well-established, it’s crucial to highlight that many NICUs primarily screen for postpartum depression (PPD), often assuming that certain questions indirectly address anxiety. Our study challenges this assumption, emphasizing the distinct and significant impact of both anxiety and depression on parental mental health.

Having uncovered nuanced insights into the contributors of parental depression symptoms through SHAP analysis, we turn our attention to the performance aspect. Remarkably, the logistic regression model, a key focus of our study, exhibits comparable effectiveness when benchmarked against commonly used ML models. This finding aligns with broader research on depression, where logistic regression has consistently demonstrated either superior or comparable performance compared to alternative ML models (31–33). Our observation prompts consideration of two pivotal factors that contribute to this alignment. First, the richness of our dataset, encompassing a broad spectrum of variables and free from biases, ensures that optimized models consistently exhibit stable performance across diverse algorithms. All eight models achieved an area under the curve (AUC) above 0.8, suggesting that the logistic regression-based model’s performance is comparable to other common ML models. Second, the common ML models typically outperform logistic models in larger datasets. However, the comparable performance observed in our study, possibly attributed to the dataset’s size (three hundred observations), underscores the value of an easily interpretable logit model for predicting postpartum depression among NICU parents, boasting a high accuracy of 0.77.

Based on our findings, while Logistic Regression offers its own advantages and remains one of the top-performing models, it is essential to consider the broader performance metrics displayed in Figure 3. Notably, algorithms such as Naïve Bayes, XGBoost, and Artificial Neural Network demonstrate a remarkable balance between precision and sensitivity as evidenced by their notably high F-score values. This underscores their ability in effectively identifying positive cases while simultaneously minimizing both false positives and false negatives. Naïve Bayes stands out as a rapid algorithm with minimal training time, making it ideal for clinical decision support systems where speed is a crucial constraint (12). XGBoost exhibits the robust ability to mitigate overfitting issues commonly encountered in datasets (34). On the other hand, Neural Network emerges as an excellent choice when dealing with substantial amounts of data sourced from diverse healthcare organizations (35). By highlighting the strengths and distinct qualities of each ML technique in relation to predicting PPD in the NICU, our study expands the potential for accurate predictions, enhances the understanding of PPD risk factors, and provides valuable insights for developing targeted interventions in NICU settings.

It is essential to acknowledge the limitations inherent in this study. Notably, the dataset under consideration exhibited a relatively small number of patients. While the size of our sample is limited, it is imperative to underscore the high quality of the data therein. This dataset originates from a meticulously conducted clinical trial, ensuring a high standard of data integrity. It is crucial to emphasize that the sample is devoid of biases, and further enhances its reliability by maintaining a balanced representation across various racial groups which instill confidence in the validity of our study outcomes. To address potential challenges associated with small sample sizes, rigorous monitoring of learning curves for all prediction models was undertaken throughout the training process. Employing a strategic combination of techniques, including cross-validation, regularization, and hyperparameter tuning, we actively mitigated the risk of overfitting, thereby reinforcing the integrity of our study’s analytical approach. Another limitation of this study is that the dataset utilized was exclusively sourced from the Children’s National Hospital in Washington, DC. Therefore, the generalizability of our study’s results to other healthcare systems monitoring parental depression symptoms in the NICU may be limited. Future studies should aim to include larger and more diverse datasets from multiple institutions to enhance the external validity and generalizability of the predictive models developed in this research.

## Conclusion

In conclusion, the findings of this study contribute to the ongoing efforts of improving parental depression screening in the NICU context. The implementation of more accurate and targeted screening systems can ease the burden on both patients and healthcare systems by reducing unnecessary interventions and optimizing resource allocation. Our findings emphasize the importance of evaluating perceived stress, perceived social support, and state anxiety scale as essential factors to be screened in NICU parents. Moreover, our results show that the performance of the logistic regression as an interpretable and easy to use model is comparable with other commonly used ML-based models. This finding facilitates informed decision-making for healthcare providers, empowering them to select the most appropriate model for their specific contexts. These advancements aim to enhance the overall well-being of parents and their infants in the NICU by effectively identifying and addressing parental depression.

## Data availability statement

The data analyzed in this study is subject to the following licenses/restrictions: this is the analysis of an existing dataset. We obtained the de-identified data from authors of this paper. Requests to access these datasets should be directed to LS, LSoghier@childrensnational.org.

## Ethics statement

This study was approved by the Children’s National Institutional Review Board and it was registered with https://clinicaltrials.gov (NCT02643472). The studies were conducted in accordance with the local legislation and institutional requirements. Written informed consent for participation was not required from the participants or the participants’ legal guardians/next of kin in accordance with the national legislation and institutional requirements.

## Author contributions

FS: Conceptualization, Formal analysis, Methodology, Visualization, Writing – original draft, Writing – review & editing. NH: Conceptualization, Funding acquisition, Supervision, Writing – review & editing. VA: Methodology, Writing – review & editing. LS: Data curation, Funding acquisition, Writing – review & editing.
